# DNMT3A mutation leads to leukemic extramedullary infiltration mediated by TWIST1

**DOI:** 10.1186/s13045-016-0337-3

**Published:** 2016-10-10

**Authors:** Jie Xu, Wu Zhang, Xiao-Jing Yan, Xue-Qiu Lin, Wei Li, Jian-Qing Mi, Jun-Min Li, Jiang Zhu, Zhu Chen, Sai-Juan Chen

**Affiliations:** 1 State Key Laboratory for Medical Genomics, Shanghai Institute of Hematology, Rui-Jin Hospital affiliated to Shanghai Jiao Tong University School of Medicine, 197 Rui-Jin Er Road, Shanghai, 200025 China; 2 Department of Hematology, the First Hospital of China Medical University, Shenyang, China; 3 Division of Biostatistics, Dan L. Duncan Cancer Center, Baylor College of Medicine, Houston, TX USA; 4 Department of Molecular and Cellular Biology, Baylor College of Medicine, Houston, TX USA; 5 Department of Bioinformatics, School of Life Sciences and Technology, Tong-Ji University, Shanghai, China

**Keywords:** *DNMT3A* mutation, Acute myeloid leukemia, Extramedullary infiltration, TWIST1

## Abstract

**Background:**

*DNMT3A* mutations are frequently discovered in acute myeloid leukemia (AML), associated with poor outcome. Recently, a relapse case report of AML extramedullary disease has showed that AML cells harboring *DNMT3A* variation were detected in the cerebral spinal fluid. However, whether a causal relationship exists between *DNMT3A* mutation (D3Amut) and extramedullary infiltration (EMI) is unclear.

**Methods:**

We took advantage of *DNMT3A* (R882C) mutation-carrying AML cell strain, that is, OCI-AML3, assessing its migration ability in vitro and in vivo. By RNA interfering technology and a xenograft mouse model, we evaluated the effect of *DNMT3A* mutation on cell mobility and explored the possible mechanism.

**Results:**

OCI-AML3 displayed extraordinary migration ability in vitro and infiltrated into meninges of NOD/SCID mice after intravenous transfusion. We found that this leukemic migration or infiltration capacity was significantly compromised by the knockdown of DNMT3A mutant. Notably, TWIST1, a critical inducer of epithelial–mesenchymal transition, which underlies the metastasis of carcinomas, was highly expressed in association with R882 mutations. Abrogation of TWIST1 in *DNMT3A* mutated cells considerably weakened their mobility or infiltration.

**Conclusions:**

Our results demonstrate that D3Amut in OCI-AML3 strain enhances leukemic aggressiveness by promoting EMI process, which is partially through upregulating TWIST1.

**Electronic supplementary material:**

The online version of this article (doi:10.1186/s13045-016-0337-3) contains supplementary material, which is available to authorized users.

## Background

Acute myeloid leukemia (AML) is a group of subtypes that share common features with various manifestations. Extramedullary infiltration (EMI) is a specific symptom of bone marrow diseases, such as myeloid sarcoma, leukemia cutis, and central nervous system (CNS) leukemia. The prognosis of extramedullary event is controversial but generally considered an advanced malignancy and indicator of poor outcome [1, 2]. The mortality rate caused by EMI, to some extent, is reduced by the means of standard systemic chemotherapy combined with local treatment, such as intrathecal injection and skin radiation [3]. However, extramedullary relapse after chemotherapy, even hematopoietic stem cell transplantation, is still common [4, 5].

Several lines of clinical analyses demonstrated that the patients with abnormal karyotypes, such as t (8; 21), inv (16), and 11q23 translocations, tend to have extramedullary diseases [1]. With regard to immunophenotype, CD56-positive leukemic cells are prone to infiltrate [6]. Additionally, a family of matrix metalloproteinases (MMPs) is considered to facilitate cell invasion into soft tissues and CNS [7–9]. This evidence confirms that molecular markers are useful to predict leukemic progressive invasiveness.

Recently, a case report on an AML-M2 patient relapsed with CNS leukemia after achieving complete remission (CR) has attracted attention. Although no *DNMT3A* mutation (D3Amut) is detected in the bone marrow and her buccal mucosal cells at diagnosis, deletion of exon 18 in *DNMT3A* is observed in the cerebral spinal fluid (CSF) on relapse stage [10]. However, the mechanism on how the chemo-resistant subclone with D3Amut could emerge in CNS remains unknown.

Mutated *DNMT3A* is highly relevant to higher WBC counts, older age, and shorter survival in AML with mutations compared with those with wild-type (WT) *DNMT3A* [11, 12]. Mutated *DNMT3A* occurs in hematopoietic stem cells and is considered a driver mutation in initiating leukemia [13]. D3Amut is relatively obstinate. It can persist in cases with morphologically CR [14] and be closely associated with disease relapse or progression [15, 16]. Interestingly, this mutation has been frequently identified in myelomonocytic and monoblastic phenotypes of AML (AML-M4/M5) [11]. With these two subtypes, patients are more likely to have EMI presentation [2, 17]. Nevertheless, whether D3Amut takes part in EMI process is unclear.

In the present study, D3Amut could promote cell migration. OCI-AML3, a leukemia cell line harboring the hotspot *DNMT3A* R882C mutation [18], could proliferate in NOD/SCID mice and induce paralysis and finally death. Paralysis symptom was mentioned in a previous study [19]. Our investigation demonstrated that this particular symptom is caused by murine CNS leukemia, which could be attributed to the cells bearing D3Amut. Intriguingly, an epithelial–mesenchymal transition (EMT) inducer, TWIST1, is activated upon D3Amut and could facilitate aberrant leukemic cell migration.

## Methods

### Leukemic cell lines

Human AML cell lines (OCI-AML3, Kasumi-1, NB4, THP-1, and U937) were all suspended and cultured in RPMI-1640 medium (Invitrogen, Grand Island, USA) with 10 % FBS (Invitrogen, Grand Island, USA). OCI-AML3 strain was kindly provided by Dr. Lan Wang (Shanghai Institutes for Biological Sciences, China). The four other cell lines were obtained from Shanghai Institute of Hematology. Logarithmically growing cells were used for the experiments.

### Primary AML blasts

Total bone marrow cells were collected from diagnosed AML patients. These fresh cells were immediately purified via density gradient centrifugation using Ficoll. Leukemia blasts were harvested in the mononuclear layer for experiments or storage. All patients provided written informed consent for the use of their AML samples under a protocol approved by the ethics committee of Shanghai Institute of Hematology. Human primary AML samples were obtained in accordance with the ethical guidelines established by Shanghai Institute of Hematology.

### AML mouse model

Human AML cell strains OCI-AML3, U937, and THP-1 with or without exogenous plasmids transduction were prepared in about (1–10) × 10^6^ number. Cells were injected into lethally irradiated 8-week-old NOD/SCID mice through tail veins. Around 1 month post xenografting or at the time of paralysis, leukemic cells in murine peripheral blood, bone marrow, spleen, or brain were examined. All animal experiments were carried out in accordance with the approved guidelines provided by the Laboratory Animal Resource Center of Shanghai Jiao Tong University School of Medicine.

### RNA interference

The procedures of siRNA transfection and lentivirus-mediated shRNA infection were described previously [20]. The sequences of *DNMT3A* siRNA oligomers and shRNA primers were according to previous study [20]. The sequences of human *TWIST1* siRNA oligomers were as follows:

siTWIST1-1:

- 5′-UCUAAUUUCCAAGAAAAUCUU-3′ (forward),
- 5′-GAUUUUCUUGGAAAUUAGAAG-3′ (reverse);
- siTWIST1-2:
- 5′-AGUAUUUUUAUUUCUAAAGGU-3′ (forward),
- 5′-CUUUAGAAAUAAAAAUACUGG-3′ (reverse).
- The primers for human *TWIST1* shRNA were as follows:
- sh-TWIST1-1: 5′-CCGGGCTGGACTCCAAGATGGCAAGCTCGAGCTTGCCATCTTGGAGTCCAGCTTTTTG-3.
- sh-TWIST1-2: 5′-CCGGAAGATTTTCTTGGAAATTAGACTCGAGTCTAATTTCCAAGAAAATCTTTTTTTG-3′.

### Real-time quantitative PCR

cDNA templates were prepared after RNA extraction and reverse transcription. Amplification was performed on a real-time PCR system (Applied Biosystems 7500, USA). The whole procedure was according to the manual of SYBR^®^ Premix Ex Taq™ kit (Takara RR420A, Japan). Relative expression was calculated using the formula of 1/2^△△Ct^.

### Transwell assay

Transwell^®^ Permeable Supports (Costar 3421, Corning, USA) was prepared to test cell migration. The assay was carried out following the protocol provided by the manufacturer.

### Scratch-wound assay

About 6-cm diameter dish (Falcon, Bedford, USA) was fully grown with adherent cells, and DMEM (Invitrogen, Grand Island, USA) medium with 10 % FBS was replaced with pure DMEM medium. Cells were starved for 16 h in an incubator at 37 °C and 5 % CO_2_. Subsequently, the monolayer cells in the middle of the dish were scratched using a sterile tip, and the dish was continuously incubated for 12 h. Finally, cell number in the wound was observed using a microscope (Nikon, TS100, NY, USA).

### Flow cytometry and cell sorting

Immunophenotyping assays were all analyzed on LSRII Flow Cytometer (Franklin Lakes, NJ, USA). Flow data were further analyzed by FlowJo software (TreeStar, Ashland, OR, USA). Antibodies were as follows: human CD44-APC and human CD45-PE (BD Pharmingen™, NJ, USA). Leukemic cells carrying green fluorescence proteins (GFPs) or red fluorescence proteins were detected and selected by MoFlo flow-sorter (Beckman coulter, Fullerton, CA, USA).

### Western blotting

SDS-PAGE gels were prepared depending on protein size. Electrophoresis and transmembrane were carried out on a protein electrophoresis and blotting system (Bio-Rad, Hercules, CA, USA). The signals were visualized using a chemiluminescence detector (LAS-4000, FUJIFILM). The antibodies used in this study were DNMT3A, SNAIL, Flag, GAPDH (Cell Signaling, Danvers, USA), TWIST1, VIMENTIN (Santa Cruz, Santa Cruz, USA), and β-actin (Sigma, St. Louis, MO, USA).

### Morphological analysis

Bone marrow cytospins and sections were subject to Wright–Giemsa staining for morphological analyses. Leukemic cells with Wright–Giemsa staining are large and with round shape, pale blue cytoplasm, and pink nucleus.

### Immunohistochemical staining and immunofluorescence

Murine brain sections and spinal cord slices were prepared for HE staining and human CD44 (Santa Cruz) immunohistochemical staining. Exogenous cells labeled by hCD44 in CNS were surrounded with brownish red color. Murine brain or BM slices were observed under Leica TCS SP8 confocal microscope (Leica Microsystems, Wetzlar, Germany) after incubation with GFP (Cell Signaling) or human CD44 antibody.

### PET-CT

Around 1 month post xenografting, paralyzed mice transplanted with OCI-AML3 strains were used for PET-CT scanning (Siemense, Inveon PET-CT, USA) after injecting fluorodeoxyglucose into mice bodies via tail veins. Except for the brain, heart, and bladder, the sites presented in highlight indicated a concentration of leukemic cells caused by their active metabolism.

### Bioluminescence imaging

Human AML cells carrying luciferase reporter were transplanted into NOD/SCID mice. Luciferase substrate was injected into living animals before imaging. In vivo imaging system (Xenogen IVIS Spectrum, PerkinElmer) was used for catching the fluorescence from the whole body.

## Results

### DNMT3A R882 mutation enhances cell migration in vitro

**Fig. 1:**
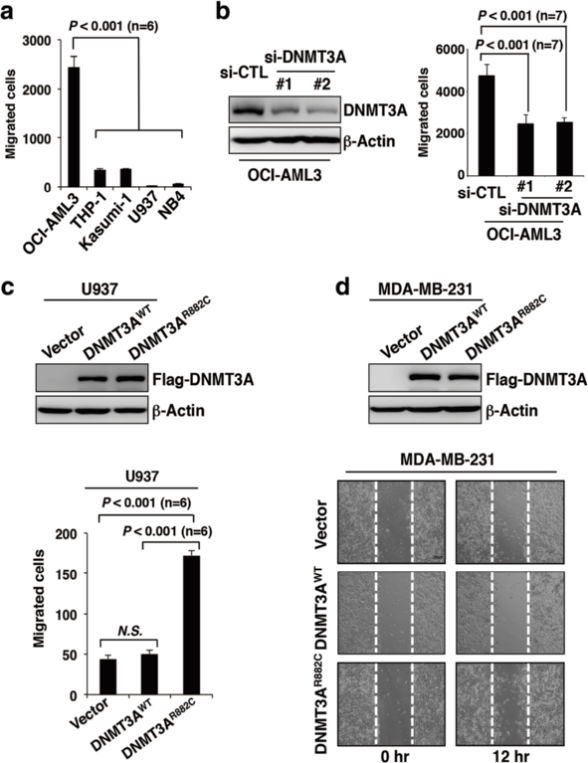
DNMT3A R882 mutation enhances cell migration in vitro*.* **a** Transwell assays of acute leukemia cell lines OCI-AML3, THP-1, Kasumi-1, U937, and NB4. Each strain is seeded in a number of 1 × 10^4^ cells. Data are expressed as mean ± SD; *n* = 6 per group. **b** Transwell assays of OCI-AML3 with or without *DNMT3A* mRNA knockdown. About 1 × 10^4^ of cells are purified for inoculation. Expression levels of DNMT3A proteins are shown in the *left panel*. Data are presented as mean ± SD for each group (*n* = 7). **c** Transwell assays of U937 cells that are stably transfected by lentivirus with wild-type (WT) DNMT3A or DNMT3A R882C. Overexpressed WT and mutant DNMT3A are indicated by Flag antibody in the *upper panel*. Approximately 1 × 10^4^ of purified cells is used for plating in each well. Data are expressed as mean ± SD (*n* = 6 per group). **d** Scratch-wound assays of MDA-MB-231 cells stably expressing the vector of WT DNMT3A or DNMT3A R882C tagged by Flag. *Upper panel* shows the exogenous DNMT3A expression by anti-Flag. Representative images in lower panel are shown at 0 and 12 h after scratching

We collected five AML cell lines (OCI-AML3, THP-1, Kasumi-1, U937, and NB4) to examine their migration capacity by using transwell assay. After 18 h of incubation, the number of migrated cells of OCI-AML3 was higher than those of four other cell lines (*p* < 0.001) (Fig. 1a). OCI-AML3 cells harbor D3Amut and *NPM1* abnormality. *NPM1* mutation is considered a marker for relatively good prognosis, whereas D3Amut is associated with poor outcome. Therefore, we focused on the possibility of enhanced invasion of malignant cells rendered by *DNMT3A* R882 mutation, which is an established poor prognosis indicator. The protein level of DNMT3A in OCI-AML3 was not the highest among the five cell lines investigated (Additional file 1: Figure S1a). Subsequently, all exons of *DNMT3A* gene were sequenced in the five lines, and the results showed the existence of D3Amut only in OCI-AML3 (Additional file 1: Figure S1b). We further constructed two *DNMT3A* knockdown sublines of OCI-AML3 via specific siRNAs. The silence of *DNMT3A* could dramatically reduce the capacity of cell migration compared with control siRNA (*p* < 0.001) (Fig. 1b). To rule out the effect caused by WT DNMT3A, *DNMT3A* mRNA was downregulated in U937 cell line, which was derived from a monocytic leukemia case without D3Amut (Additional file 1: Figure S1b and S1c). Transwell assay showed that, with WT DNMT3A decreasing, migration capacity of U937 was not significantly altered (Additional file 1: Figure S1c).

To further assess the effect of D3Amut on cell migration, we overexpressed the WT and mutant DNMT3A in U937, which exhibited low migration capacity (Fig. 1a, c). Repeated independent transwell assays showed that the numbers of migrated U937 cells stably expressing DNMT3A R882C were higher than those without D3Amut (*p* < 0.001) (Fig. 1c). In addition to leukemia cells in suspension culture, the effect of D3Amut on migration behavior in adherent cells was also investigated. To eliminate the possible effect of endogenous DNMT3A protein, we tested a series of solid tumor cell lines and found little DNMT3A expression in MDA-MB-231, a breast cancer cell line (Additional file 1: Figure S1d). Scratch-wound experiment was conducted, and cell motility was efficiently increased in MDA-MB-231 cells expressing D3Amut but not in those expressing WT DNMT3A (Fig. 1d). Taken together, these data suggest that *DNMT3A* R882 mutation contributes to enhance the migration of malignant cells.

### EMI of OCI-AML3 cells in mice

**Fig. 2:**
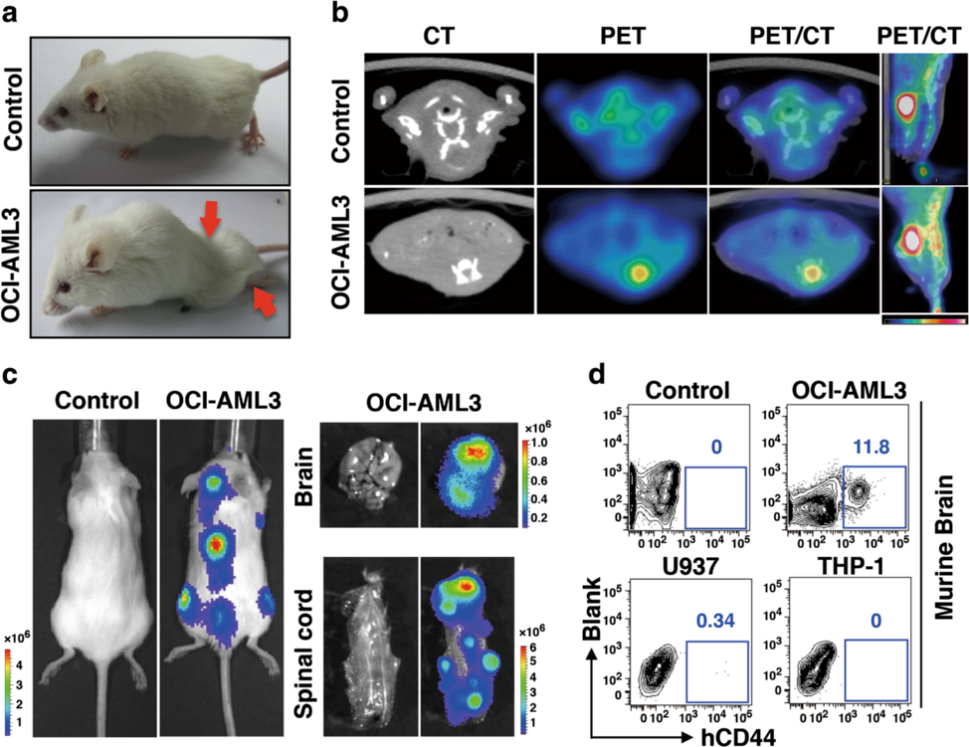
Extramedullary infiltration of OCI-AML3 cells in mice. **a** Representative NOD/SCID mouse transplanted with OCI-AML3 cell line presents paralysis syndrome at 1 month post xenografting. *Red arrows* indicate the paralyzed hind limbs. **b** PET and CT images show leukemia cells infiltration in murine spinal cords. Radioactive substrates distributed in bladders are shown as the internal controls. **c** Bioluminescent imaging of NOD/SCID mice at 1 month post xenografting. OCI-AML3 cells with luciferase are distributed mainly in the brain, spinal cord, and BM. **d** Representative FACS plots of digested cells from control and OCI-AML3-, U937-, and THP-1-xenografted murine brains, where human CD44-positive (hCD44^+^) human sources cells are gated to indicate

To evaluate the migration capacity of OCI-AML3 cell line in vivo, we transplanted OCI-AML3 cells into semi-lethally irradiated NOD/SCID mice through tail veins. Different quantities of OCI-AML3 cells, namely, 1 × 10^6^, 5 × 10^6^, and 1 × 10^7^ cells, were inoculated into mice. Around 20 days after xenografting, 36 of 37 animals investigated developed weakness in hind limbs and walking in unequal steps, with the symptoms appearing the earliest in mice inoculated with 1 × 10^7^ cells, followed by those with 5 × 10^6^ and 1 × 10^6^ cells (Fig. 2a). X-ray examination eliminated the possibility of pathological bone fracture in their hind limbs (Additional file 1: Figure S2a). Mice were executed when they became moribund. The average survivals in the three groups challenged with OCI-AML3 cells were 20.6, 23.9, and 30.0 days (*p* < 0.001 compared with the controls) (Additional file 1: Figure S2b). Mild splenomegaly was observed in sick mice. Immunophenotypic staining of human leukocyte-specific antigens hCD45 and hCD44 revealed the presence of OCI-AML3 cells in spleens (Additional file 1: Figure S2c), and the percentage of exogenous leukemia cells in the bone marrow (BM) was relatively low (Additional file 1: Figure S2c).

To explore the cause of paralysis presentation, we proposed the possibility of CNS abnormality caused by extramedullary leukemia. Positron emission tomography-computed tomography (PET-CT) was performed to investigate the hypermetabolic regions that are suggestive of tumor infiltration. Compared with those of normal controls, the spinal cords of sick mice contained more radioactive substances (Fig. 2b), supporting the infiltration of OCI-AML3 cells into CNS. To detect the disease status at the entire organism level, we transfected luciferase reporter lentivirus vector into OCI-AML3 cells and subsequently transplanted the cells into the mice. All transplanted animals developed a paralysis syndrome at 1 month post xenografting. Xenografts were observed to infiltrate the brains, spinal cords, and BMs by using bioluminescent imaging (Fig. 2c). Flow cytometry showed quite a lot of xenografted cells in the brain when hCD44 antigens were labeled (Fig. 2d). To examine whether other leukemia cell lines could cause CNS infiltration, we used 5 × 10^6^ cells of U937 and THP-1 lines, both from acute monocytic leukemia patients, for transplantation. Around 20 days post xenografting, U937 cell-transplanted mice developed severe leukemia, but few exogenous cells were detected in the brain (Fig. 2d). In THP-1 cell-transplanted mice, no leukemia infiltration was observed in cerebral tissues at 40 days post xenografting when the mice became moribund (Fig. 2d). The above phenomenon demonstrates that OCI-AML3 cells could infiltrate into murine CNS and induce characteristic clinical symptoms.

### CNS-infiltrating leukemic cells concentrate in murine meninges

**Fig. 3:**
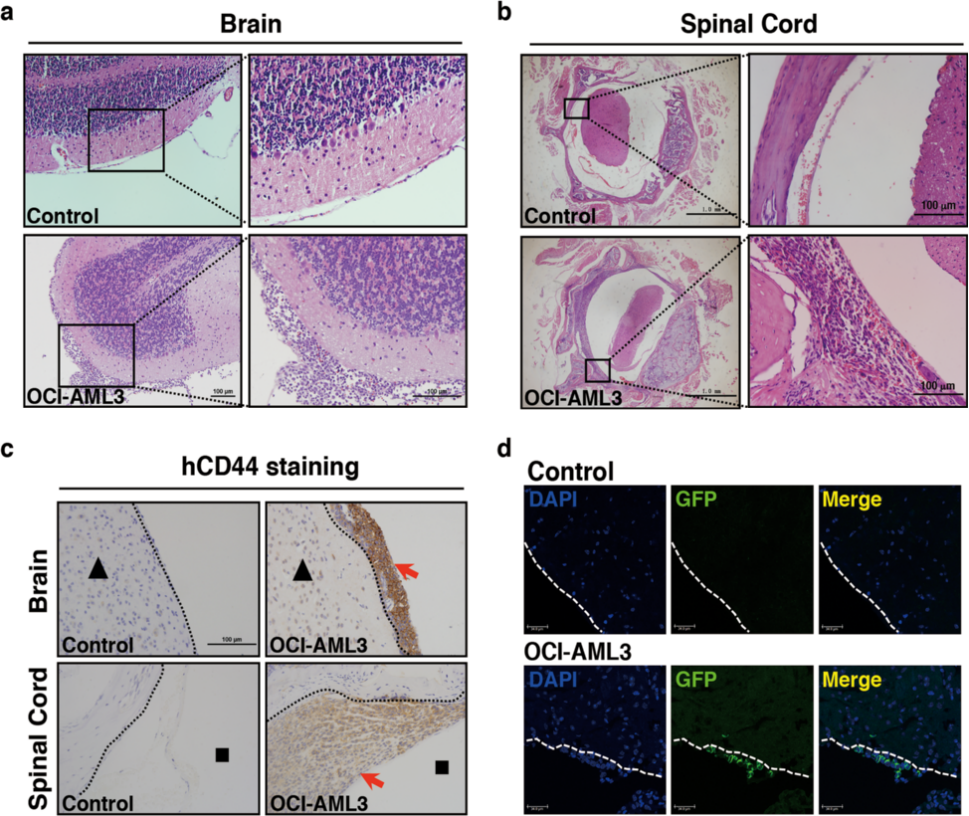
CNS-infiltrating leukemic cells concentrate in murine meninges. **a** HE staining of brain biopsies taken from control or transplanted mice. Meningeal spaces are boxed in *black*. Myeloid cells infiltrate into brain meninges of OCI-AML3 group. **b** HE staining of spinal cord biopsies from control or transplanted mice. *Enlarged black boxes* represent spinal meninges. Xenografts distributed along the spinal meninges are detected in transplanted mice. **c** Immunohistochemical staining of murine brain and spinal cord. hCD44^+^ cells in *brown*, pointed by *red arrows*, indicate the OCI-AML3 cells in transplanted mice. Black triangles and squares denote cerebral parenchymas and spinal cavities, respectively. *Dashed lines* indicate the meninges. **d** Immunofluorescence shows murine brain meninges infiltrated by OCI-AML3 labeled by GFP antibody

Histopathologic examinations were carried out to further verify the CNS-infiltrating characteristics. In sick mice tissues, myeloid leukemia cells with large and round shape, pale cytoplasm, and poly or band form nucleus were accumulated in the brain meninges (Fig. 3a) and spinal cord meninges (Fig. 3b). Immunohistochemistry results showed that these cells were also positive for hCD44 (Fig. 3c). When mice transplanted with GFP vector-containing OCI-AML3 cells were examined, GFP-positive cells were shown along the brain meninges (Fig. 3d). HE staining and immunofluorescence showed the presence of around 10 % of exogenous leukemia cells in murine BMs (Additional file 1: Figure S3a and S3b). In addition, the genetic lesions of R882 loci were detected to validate the exogenous cells. Targeted sequencing results showed that GFP-positive samples from BMs and brains all harbored R882C mutation, which was the same as the control cells (Additional file 1: Figure S3c). Thus, OCI-AML3 cells are verified to invade the brain and spinal cord meninges and induce CNS leukemia.

### Abrogation of D3Amut exerts an anti-infiltration effect

**Fig. 4:**
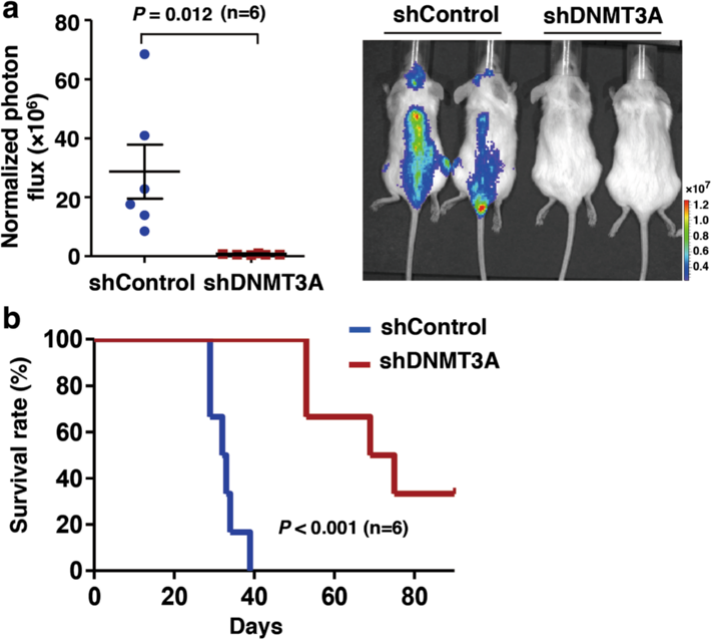
Abrogation of D3Amut exerts an anti-infiltration effect. **a** Quantitative luciferase bioluminescence is monitored at day 30 post xenografting. Values are as mean ± SEM (*n* = 6 per group). Representative bioluminescent image of mice transplanted with OCI-AML3 cells stably expressing control shRNA (shControl) or shRNA-targeting *DNMT3A* (shDNMT3A). **b** Kaplan-Meier analysis shows the survival rates of mice receiving OCI-AML3 cells stably expressing a non-targeting shControl or an shDNMT3A

Leukemic CNS infiltration indicates poor clinical outcome. In this study, mice with a large number of leukemia cells that infiltrated the meninges tended to have worse general status. OCI-AML3 cells could infiltrate into CNS in vivo, and D3Amut could promote cell migration in vitro; hence, we speculated that inhibition of heterozygous D3Amut in OCI-AML3 cells could affect the ability of leukemic cell migration in mice. A non-targeting control shRNA (sh-CTL) and two shRNAs targeting *DNMT3A* (sh-DNMT3A-1 and 2) were stably transfected into OCI-AML3. In these two knockdown strains, cells with sh-DNMT3A-2 were collected for transplantation because the expression of DNMT3A was more reduced than that in other strain (Additional file 1: Figure S4). Thus, OCI-AML3 cells with sh-CTL and sh-DNMT3A-2 were purified in a number of 1 × 10^6^ cells for xenograft in NOD/SCID mice. A luciferase reporter lentivirus vector was subsequently brought into OCI-AML3-sh-CTL (shControl) and OCI-AML3-sh-DNMT3A-2 (shDNMT3A) cells. With the same numbers of transplanted cells, the motility of OCI-AML3 with reduced D3Amut was largely decreased in the brains and spinal cords of mice at 1 month post xenografting (Fig. 4a). Notably, mice inoculated with shDNMT3A cells had prolonged life spans than those with shControl cells (*p* < 0.001) (Fig. 4b). With regard to the mortality in the shControl group, the contamination of a small number of OCI-AML3 cells without DNMT3A knock down might compete against shDNMT3A to cause late leukemic explosion. These data show that abrogation of D3Amut could exert an anti-infiltration effect and reduce the invasiveness of leukemic cells.

### TWIST1 represents a downstream target of D3Amut

**Fig. 5:**
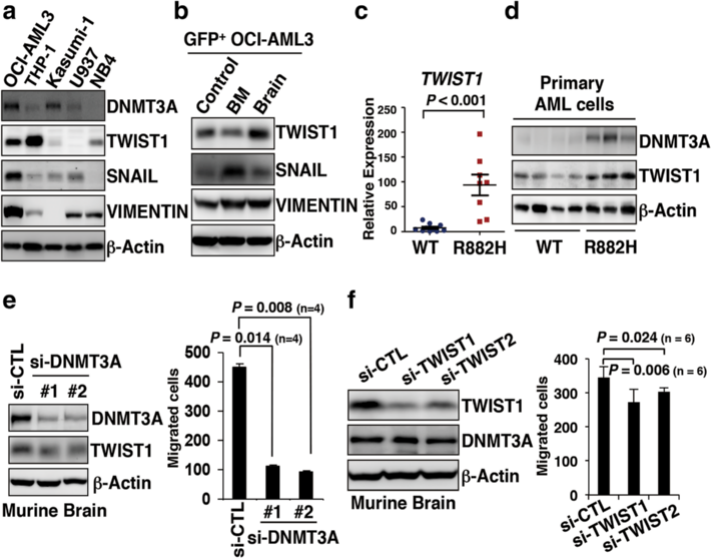
TWIST1 represents a downstream target of D3Amut. **a** Western blot of DNMT3A and EMT markers in acute leukemia cell lines OCI-AML3, THP-1, Kasumi-1, U937, and NB4. **b** Western blot presents the EMT markers in GFP-positive OCI-AML3 strains and those sorted from murine BMs and brains via GFP tags. **c** Real-time PCR of the mRNA levels of *TWIST1* in AML patients’ primary BM cells with DNMT3A WT and DNMT3A R882H mutations. Bar graphs show the mean ± SD of ten and eight cases in WT and mutant groups, respectively. **d** Western blot shows the DNMT3A and TWIST1 in AML patients’ primary BM cells with DNMT3A WT and DNMT3A R882H mutations. **e** *Left panel* shows the Western blot analysis of TWIST1 and DNMT3A in sorted OCI-AML3 cells located in murine brain. Those cells are transiently transfected by scramble siRNA (si-CTL) or two different siRNAs targeting *DNMT3A* mRNA (si-DNMT3A-1 and 2). Transwell assays of OCI-AML3 cells from murine brain with or without *DNMT3A* mRNA knocked down are shown in the *right panel*. About 200 purified cells are used for plating in one well. Data are presented as mean ± SD for each group (*n* = 4). **f** Western blot of DNMT3A and TWIST1 in OCI-AML3 cells sorted from murine brain and are transiently transfected by si-CTL or siRNAs targeting *TWIST1* mRNA (si-TWIST1 and si-TWIST2). Transwell assays of those sorted cells with si-CTL, si-TWIST1, or si-TWIST2. Cells are seeded in a number of 1 × 10^4^. Data are expressed as mean ± SD (*n* = 6 per group)

To obtain insight into how D3Amut caused aberrant cell migration of OCI-AML3, we analyzed a group of EMT markers in OCI-AML3 strain. EMT-associated genes promote cancer cell plasticity and tumor metastatic spread, and the process can be induced by some factors, such as TWIST1 and SNAIL [21]. We observed that OCI-AML3 cells harbored high expression levels of TWIST1, SNAIL, and VIMENTIN. In other leukemia cell lines, these EMT markers were relatively in lower expressions except TWIST1 also highly expressing in THP-1 (Fig. 5a). These markers in CNS-infiltrating leukemia cells were further analyzed after cell sorting via GFP-tag (Additional file 1: Figure S5a). TWIST1, the most important transcriptional factor of EMT process, was highly expressed in the brain tissues compared with that in BMs (Fig. 5b). Subsequently, a series of AML patients’ primary BM samples that harbor DNMT3A R882H was analyzed. Interestingly, the mRNA and protein levels of TWIST1 were higher in mutant cases compared with those of DNMT3A WT ones (Figs. 5c, d). Immunofluorescence also showed that TWIST1 was highly expressed in primary leukemic cells with D3Amut (Additional file 1: Figure S5b). In the meanwhile, TWIST1 protein level was increased in U937 or MDA-MB-231 strain by overexpression of D3Amut but not WT DNMT3A (Additional file 1: Figure S5c). Also in Kasumi-1 cells, the expressional levels of TWIST1 were not changed after WT DNMT3A silencing so as to further exclude the possible effect made by WT DNMT3A (Additional file 1: Figure S5d). To evaluate the effect of D3Amut on the expression of TWIST1, OCI-AML3 cells stably transfected with sh-DNMT3A lentivirus displayed reduced TWIST1 with silenced D3Amut (Additional file 1: Figure S4). The OCI-AML3 cells in the brain had migrating capacity; thus, DNMT3A R882C was knocked down in those cells to determine whether phenotypes could be converted. The results showed that TWIST1 expression and cell migration were both reduced in brain-infiltrating cells (Fig. 5e). TWIST1 plays a key role during EMT. Therefore, we next transiently transfected two siRNAs targeting *TWIST1* mRNA in brain-infiltrating cells. With decreasing TWIST1 level, DNMT3A remained in abundance, whereas the migration ability of brain-infiltrating cells was significantly reduced (Fig. 5f). This observation indicates that TWIST1 is probably a downstream of DNMT3A and could be involved in the regulation of leukemia cell mobility process.

### TWIST1 mediates enhanced cell migration ability in vivo

**Fig. 6:**
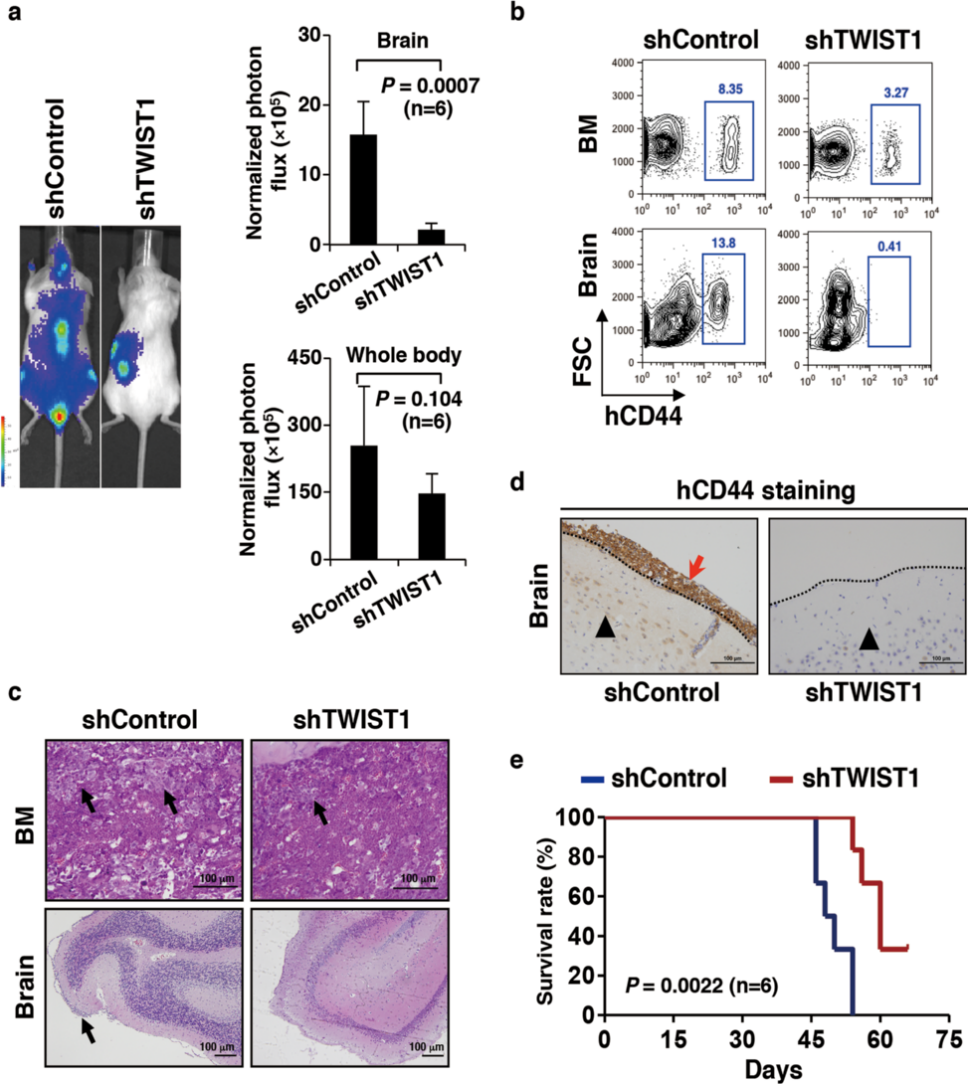
TWIST1 mediates enhanced cell migration ability in vivo*.* **a** Representative bioluminescent imaging of mice transplanted with OCI-AML3 cells carrying luciferases and stably expressing scramble shRNA (shControl) or shRNA targeting *TWIST1* (shTWIST1). Quantification of bioluminescent imaging in brains and whole bodies is shown on the *right panel*. Values are presented as mean ± SEM (*n* = 6 per group). **b** Scatter plots present the OCI-AML3 cells with or without *TWIST1* mRNA knockdown in murine BMs and brains, respectively. The percentage of exogenous cells is gated in *blue box*. **c** HE staining of BM and brain biopsies taken from shControl or shTWIST1 mice. *Black arrows* indicate leukemic cells in marrow cavities or brain meninges. **d** Immunohistochemical staining of hCD44^+^ cells indicated by *red arrow* in shControl murine brain. *Black triangles* denote cerebral parenchymas. *Dashed lines* indicate the meninges. **e** Kaplan-Meier analysis shows the survival rates of shControl and shTWIST1 mice (*n* = 6 per group)

The role of TWIST1 on the migration ability of leukemic cells in vivo was investigated. Two shRNAs (sh-TWIST1-1 and 2) targeting *TWIST1* mRNA were stably transfected into OCI-AML3 cells by lentiviruses. Western blot analysis showed that both shRNAs successfully decreased TWIST1 expressions, and the knockdown effect was more remarkable in cells with sh-TWIST1-1 compared with shControl and sh-TWIST1-2 (Additional file 1: Figure S6a). Transwell assay also revealed that migrated cells were reduced after TWIST1 knockdown, particularly by sh-TWIST1-1 interference (Additional file 1: Figure S6b). Thus, we used sh-TWIST1-1 for further study. Exogenous leukemic cells with or without stable TWIST1 knockdown (shTWIST1 or shControl) were sorted through GFP tags and transplanted into NOD/SCID mice at a same number of 5 × 10^5^. One month later, shControl mice gradually showed paralysis symptom, but such phenotype was not observed in the group of shTWIST1 mice. Bioluminescent imaging showed that in addition to BMs, exogenous grafts in control mice were also distributed in the brains and spinal cords. However, the EMI of leukemic cells in shTWIST1 mice was few in CNS regions (Fig. 6a). Flow cytometry further revealed that hCD44-positive cells were detected in BMs of both control and shTWIST1 mice. Interestingly, the analysis of brain samples showed that the control one carried quite a few leukemic cells, whereas almost no xenograft was observed in shTWIST1 mice (Fig. 6b). Similar to the data from immunophenotyping assay, histopathologic examinations with HE staining also showed that transplanted cells were obviously noted in the marrow cavities and brain meninges in control mice. By contrast, leukemic cells in the shTWIST1 group were detected in BMs but not in the brain meninges (Fig. 6c). Immunohistochemistry confirmed few hCD44-positive cells infiltrated in the brains of shTWIST1 mice (Fig. 6d). Overall survival analysis demonstrated that shTWIST1 mice had longer life spans than control ones (*p* = 0.0022) (Fig. 6e). Additionally, all shTWIST1 mice were not paralyzed in their limbs even when they died because of leukemia. The results above indicate that TWIST1 is required for meninges migration of OCI-AML3 cells in mice, and TWIST1 abrogation could significantly reduce CNS infiltration.

## Discussion

Genetic alterations are now regarded as important biomarkers for disease evaluation and prognosis assessment. In addition to chromosomal disorders, growing mutation information is recognized in EMI procedure. Some case reports found *FLT3-ITD* and *NPM1* variations in myeloid sarcomas with high frequencies of 15 and 14.4 %, respectively. Extramedullary tumors that carry these two abnormalities are mostly accompanied with cytogenetically normal AML and represent short lifespan, although *NPM1* mutation is considered a good prognostic indicator [22, 23]. This observation demonstrates that genetic mutation may independently affect disease progression in systemic leukemia with EMI.

In the present study, genetic lesion located in exon 18 of *DNMT3A* can promote leukemic cell migration. Meningeal leukemia, where EMI is displayed, could be determined in our NOD/SCID mice transplanted with human leukemic cells carrying D3Amut. Hence, we provide a strong evidence to support the clinical discovery of D3Amut in CSF from CNS relapse patient [10]. Importantly, about 20 % of our AML cases with D3Amuts and whose genetic profiles have been reported before [24] showed CNS leukemia when CSF was detected during disease courses.

D3Amuts are frequently detected in cases diagnosed with M4 or M5 subtypes of AML [25]. D3Amut alone could induce aggressive proliferation of differentiated monocytes [20], thereby suggesting that this mutation underlies the development of monocytic blasts [20]. *MLL* abnormalities, which are mutually exclusive to D3Amuts in M4/M5 variants [25], are related to extramedullary disease [6]. Our results demonstrate that D3Amut, which represents another group of AML patients with monocytic involvement, might also be associated with EMI.

The role of *DNMT3A* in cell invasion has been observed in lung cancer. Deletion of *DNMT3A* promotes tumor progression and enables cells to invade into bronchiole. Remarkably, a pool of genes in charge of cell adhesion and motion is highly expressed in *DNMT3A*-knockout mice [26]. Therefore, DNMT3A variation may enhance tumor cell invasiveness through altering migrating mechanisms [26]. In our leukemic EMI model, an EMT inducer TWIST1 is highly expressed in OCI-AML3 strains and AML patients’ bone marrow samples because of D3Amuts. EMT occurs in the initiation of metastasis for cancer progression. It enables carcinoma cells to escape cell-cell adhesion and gain migratory phenotype. EMT involvement has been experimentally proven in solid tumors [21]. Recently, a group from Italy reported that EMT-like processes are relevant to acute promyelocytic leukemia development or progression [27]. This result implicates that EMT regulator TWIST1 causes leukemia invasive behavior. Our data further suggest that the aggressive migratory behavior reminiscent of TWIST1 also exists in extramedullary leukemia and could be induced by DNMT3A R882 mutation.

DNMT3A is an epigenetic modifier, and mutation on its catalytic domain can decrease enzymatic activities and affect epigenetic modifications. We analyzed the methylation level of *TWIST1* genes in a set of primary AML samples with normal karyotype from the TCGA AML cohort [28]. This set includes 27 and 49 samples with *DNMT3A* R882 mutations and WT DNMT3A, respectively. Notably, in a region within 500-bp up- and downstream of gene transcriptional start site, R882 mutation group showed hypomethylation (Additional file 1: Figure S7). We suppose that D3Amut may lead to the demethylation of *TWIST1* gene, thereby increasing its expression in leukemic cells. Interestingly, SHI-1, a cell line harboring *MLL-AF6* translocation derived from an AML-M5 patient, can also invade murine brain [29]. *MLL* is a histone modifying gene, and *MLL* rearrangement interferes the normal function of MLL. Therefore, EMI, as one of the features of AML-M4/M5 subtypes, may be partly attributed to epigenetic deregulation.

EMI is one of the reasons for the relapsed and refractory AML. Clinical studies have demonstrated that cells bearing DNMT3A mutant are resistant to conventional chemotherapy but sensitive to high-dose of daunorubicin-based regimen [30, 31]. We suppose that dose-escalated therapy might be useful for clearance of DNMT3A mutated cells, thus disrupting cell mobility. Importantly, in our assays, abrogation of DNMT3A mutant or TWIST1 in leukemic cells reveals an anti-infiltration effect, thereby providing a possible theoretical basis for clinical transformation.

## Conclusions

In summary, our work first links D3Amut to leukemic cell migration and demonstrate that D3Amut in OCI-AML3 strain enhances leukemic aggressiveness by promoting EMI process, which is partially through upregulating TWIST1. Therefore, AML patient with this variation should be given further attention to the possibility of EMI, and D3Amut in extramedullary tumor is worth to detecting in further study. In addition, the inhibition of EMT inducer TWIST1 may be potential therapeutic target of EMI in AML.

## Additional file

Additional file 1: Figure S1. DNMT3A expression, mutational status in leukemia strains and expression level of DNMT3A in various cell lines. Figure S2. Phenotype evaluations of NOD/SCID mice transplanted with exogenous leukemic cells. Figure S3. Detection of OCI-AML3 cells in the bone marrow (BM) or brain of transplanted mice. Figure S4. Protein levels of DNMT3A and TWIST1 in OCI-AML3 with knockdowned *DNMT3A*. Figure S5. Proportion of OCI-AML3 cells in mice; expression level of TWIST1 in patient’s cells and constructed cell strains. Figure S6. TWIST1 is essential for OCI-AML3 migration. Figure S7. Compare the methylation level of *TWIST1* promoter between patients with WT and mutant DNMT3A. (DOC 3621 kb)

## Abbreviations

AML: Acute myeloid leukemia
CNS: Central nervous system
D3Amut: *DNMT3A* mutation
EMI: Extramedullary infiltration
EMT: Epithelial–mesenchymal transition
PET-CT: Positron emission tomography-computed tomography
