# Supplementary material for: DNMT3A mutation leads to leukemic extramedullary infiltration mediated by TWIST1
Source: J Hematol Oncol. 2016 Oct 10;9:106. doi: 10.1186/s13045-016-0337-3 (PMC5057205; doi:10.1186/s13045-016-0337-3)
Supplement: Additional file 1: Figure S1. — DNMT3A expression, mutational status in leukemia strains and expression level of DNMT3A in various cell lines. Figure S2. Phenotype evaluations of NOD/SCID mice transplanted with exogenous leukemic cells. Figure S3. Detection of OCI-AML3 cells in the bone marrow (BM) or brain of transplanted mice. Figure S4. Protein levels of DNMT3A and TWIST1 in OCI-AML3 with knockdowned DNMT3A. Figure S5. Proportion of OCI-AML3 cells in mice; expression level of TWIST1 in patient’s cells and constructed cell strains. Figure S6. TWIST1 is essential for OCI-AML3 migration. Figure S7. Compare the methylation level of TWIST1 promoter between patients with WT and mutant DNMT3A. (DOC 3621 kb) [file 13045_2016_337_MOESM1_ESM.doc]

**DNMT3A mutation leads to leukemic extramedullary infiltration mediated by TWIST1**

**Additional File 1**

**Figure S1.** DNMT3A expression, mutational status in leukemia strains and expression level of DNMT3A in various cell lines.

**Figure S2.** Phenotype evaluations of NOD/SCID mice transplanted with exogenous leukemic cells.

**Figure S3.** Detection of OCI-AML3 cells in bone marrow (BM) or brain of transplanted mice.

**Figure S4.** Protein levels of DNMT3A and TWIST1 in OCI-AML3 with knock-downed *DNMT3A*.

**Figure S5.** Proportion of OCI-AML3 cells in mice, expression level of TWIST1 in patient’s cells and constructed cell strains.

**Figure S6.** TWIST1 is essential for OCI-AML3 migration.

**Figure S7.** Compare the methylation level of *TWIST1* promoter between patients with WT and mutant DNMT3A.

**
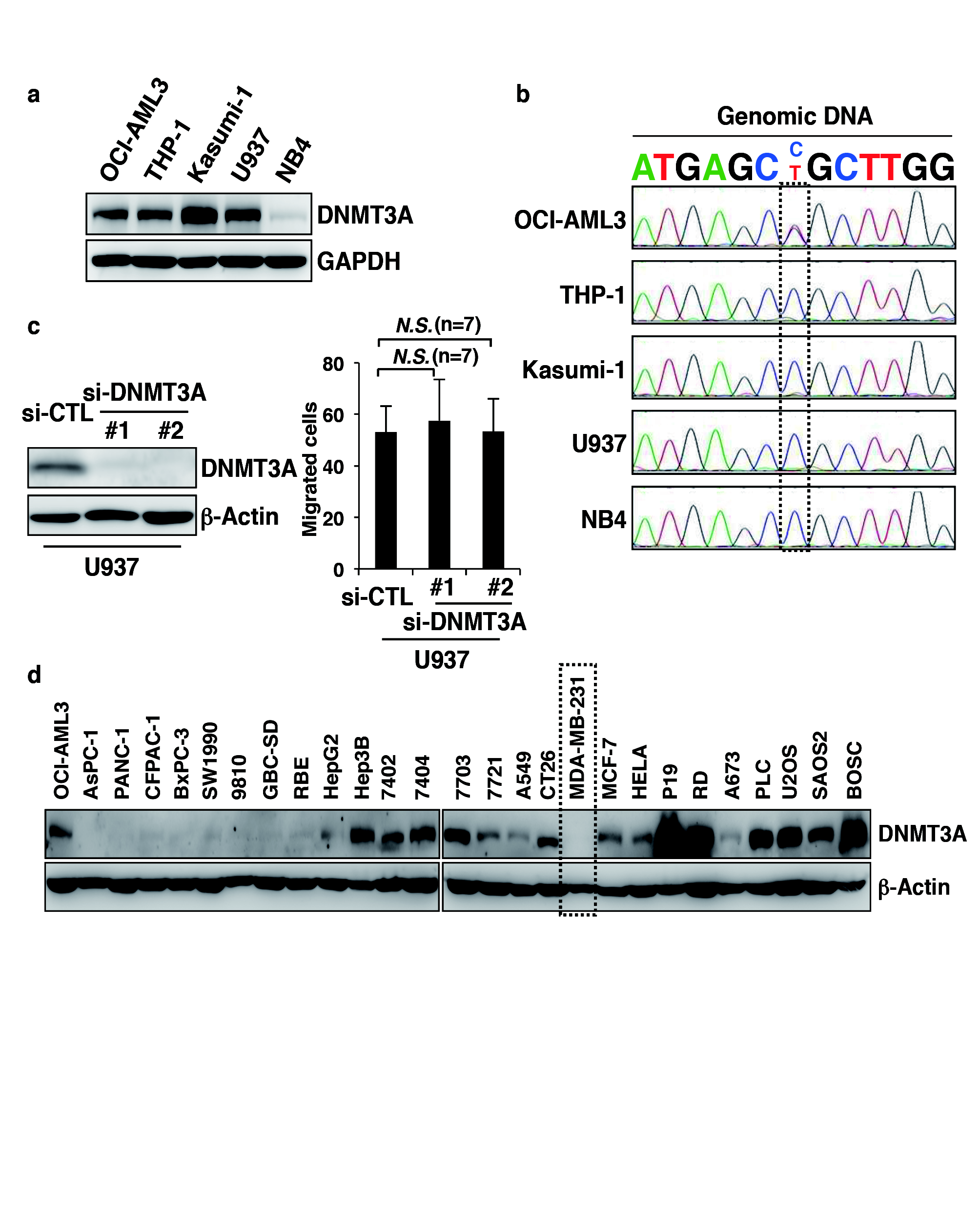
**

**Additional File 1:** **Figure S1 DNMT3A expression, mutational status in leukemia strains and expression level of DNMT3A in various cell lines.**

**(a)** Western blotting shows expression level of DNMT3A in acute leukemia cell strains OCI-AML3, THP-1, Kasumi-1, U937 and NB4. **(b)** Targeted sanger sequencing of genomic DNA extracted from OCI-AML3, THP-1, Kasumi-1, U937 and NB4. Among the detected exons of *DNMT3A*, the site at Arg882 (R882) locus is shown in dashed box. **(c)** Transwell assays of U937 with or without *DNMT3A* mRNA knockdown. About 1×10^4^ of cells are purified for inoculation. Expression levels of DNMT3A proteins are shown in the left panel. Data are presented as mean±SD for each group (n=7). **(d)** Western blotting of DNMT3A expression in 26 adherent cell lines established from multiple carcinomas or sarcomas such as pancreatic cancer, liver cancer, breast cancer, lung cancer, colon cancer. Expression of DNMT3A in OCI-AML3 is shown as positive control.


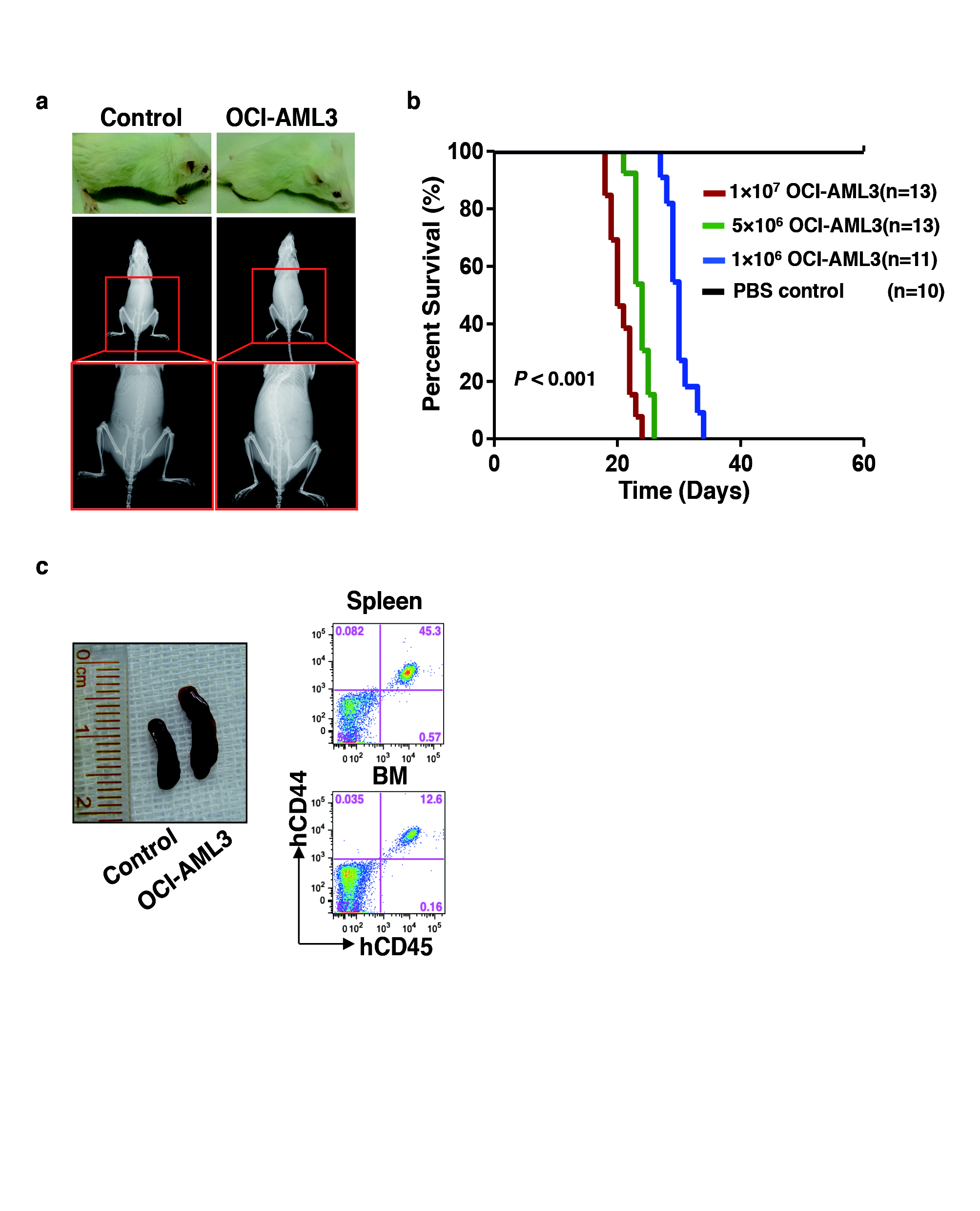


**Additional File 1:** **Figure S2 Phenotype evaluations of NOD/SCID mice transplanted with exogenous leukemic cells**

**(a)** X-rays imaging of murine bones in hind limbs of control and OCI-AML3-transplanted mice at one months post-xenografting. **(b)** Kaplan-Meier analysis of life spans in mice that receive 1×10^7^, 5×10^6^ or 1×10^6^ OCI-AML3 cells and PBS control. **(c)** The comparison in size of spleens from control and OCI-AML3-transplanted mice is shown on the left panel. Representative FACS plots presented on the right panel shows the spleen and bone marrow (BM) cells harvested from OCI-AML3-transplanted group when mice are paralyzed and moribund.

**
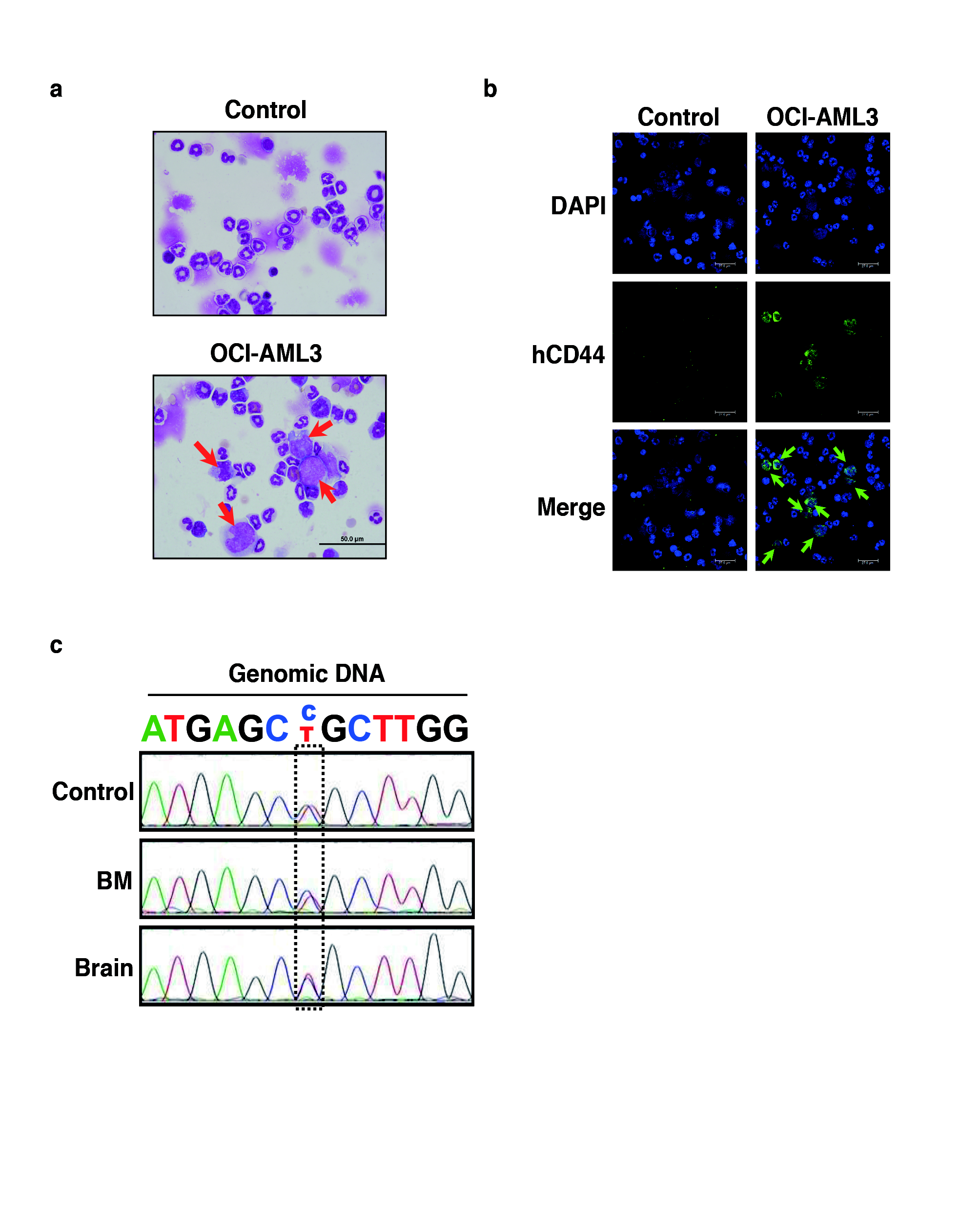
**

**Additional File 1:** **Figure S3 Detection of OCI-AML3 cells in bone marrow (BM) or brain of transplanted mice**

**(a)** HE staining of bone marrow (BM) cells in control mice and OCI-AML3 transplanted mice. Red arrows indicate the exogenous leukemia cells with big shape and loosely arranged euchromatin. **(b)** OCI-AML3 cells in murine BM are shown as hCD44 positive by immunofluorescence. **(c)** Sanger sequencing of Arg(R)882 loci in transcripts of OCI-AML3 strains (Control), OCI-AML3 cells sorted from murine BM and brain. Dashed box indicates the possible mutational position.


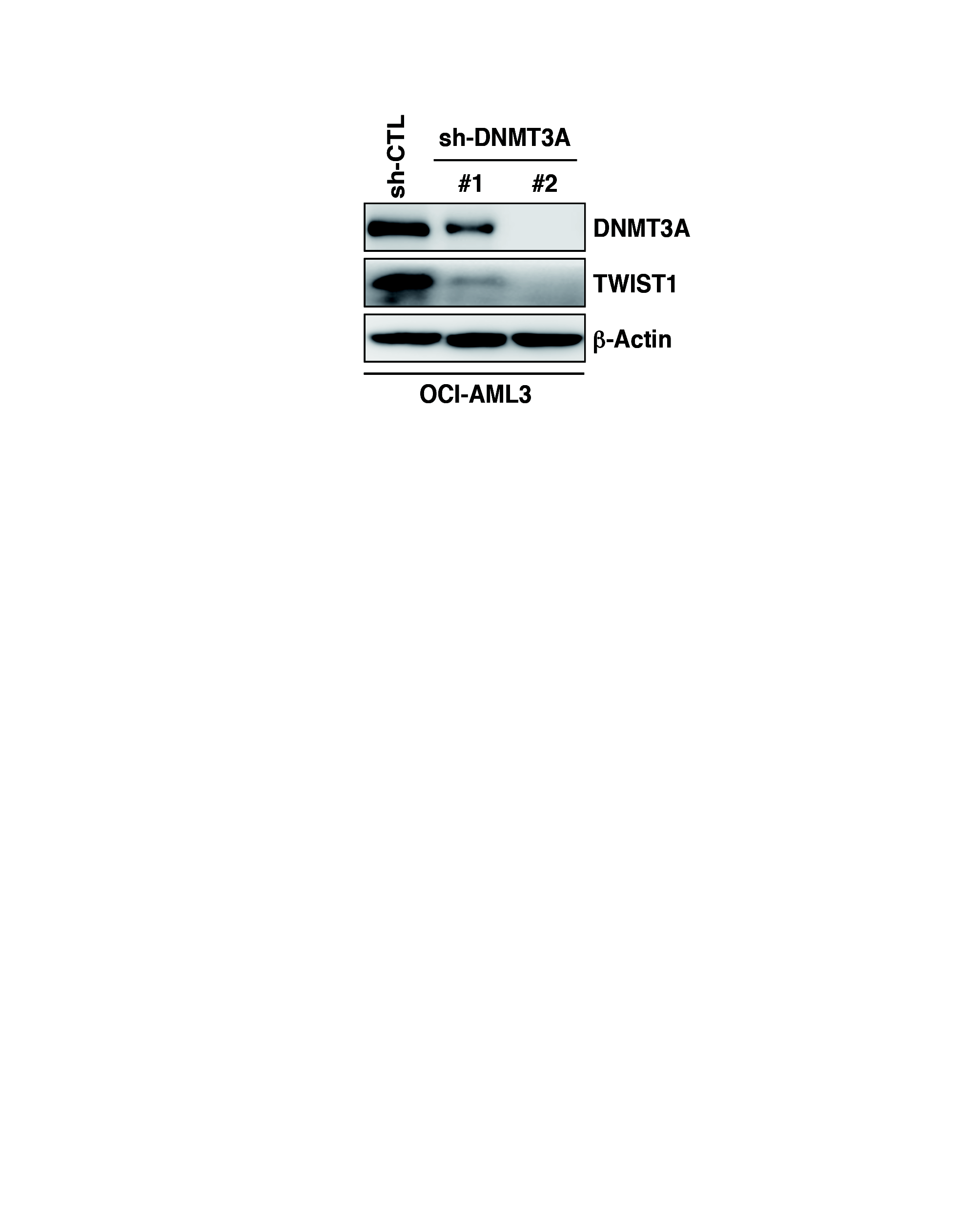


**Additional File 1:** **Figure S4 Protein levels of DNMT3A and TWIST1 in OCI-AML3 with knock-downed *DNMT3A*.**

Western blot of DNMT3A and TWIST1 in OCI-AML3 cells stably expressing a non-targeting control shRNA (sh-CTL) or two different shRNA-targeting *DNMT3A* (sh-DNMT3A#1 and #2).

**
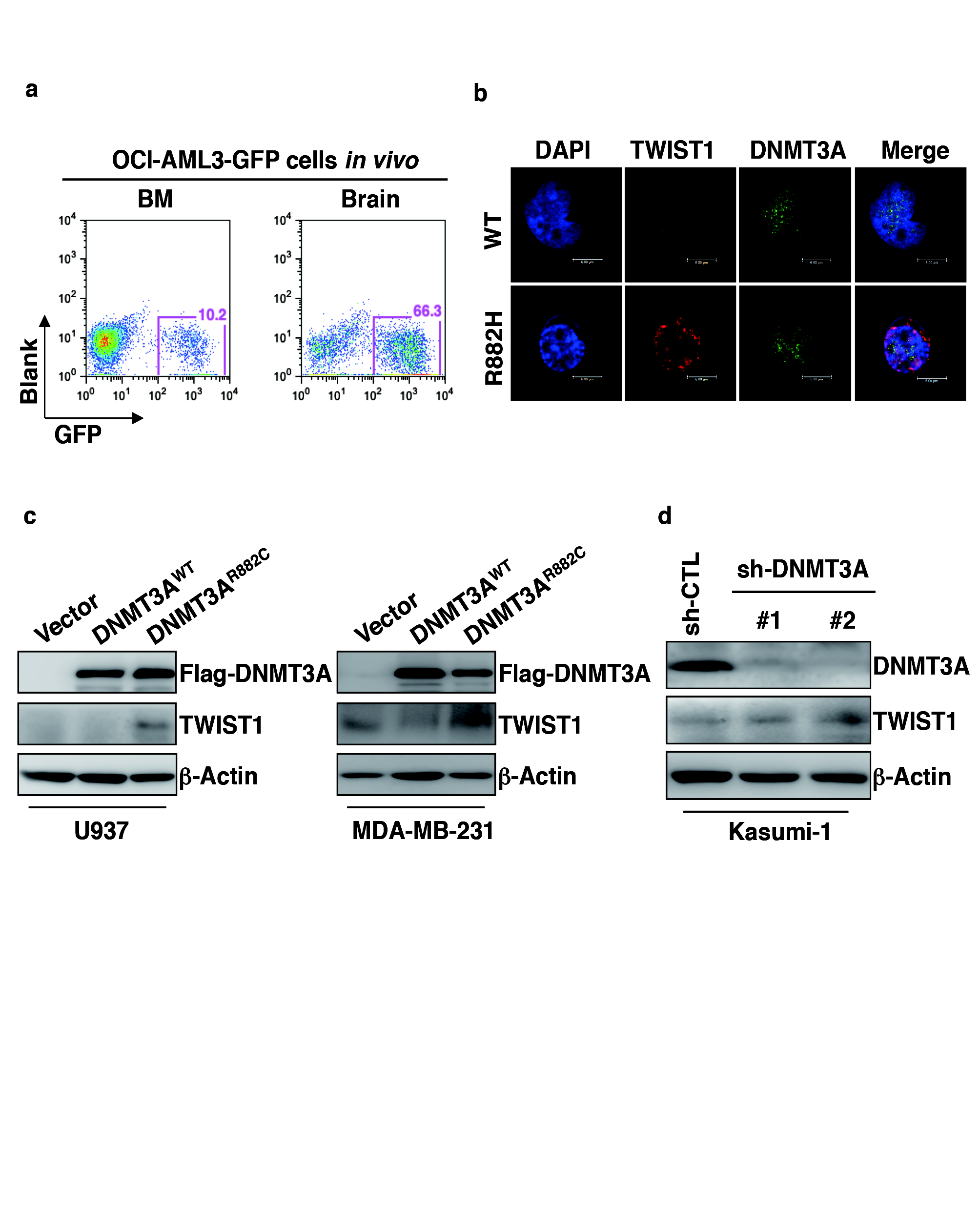
**

**Additional File 1:** **Figure S5 Proportion of OCI-AML3 cells in mice, expression level of TWIST1 in patient’s cells and constructed cell strains.**

**(a)** Scatter plots show the percentage of GFP positive OCI-AML3 cells in BM and brain of transplanted mice. The cells gated in pink box are sorted out for further study. **(b)** Immunofluorescence shows the expression of TWIST1 (red) and DNMT3A (green) in AML patients’ primary BM cells carry mutant DNMT3A or not. **(c)** Western blot of DNMT3A and TWIST1 in U937 or MDA-MB-231 cells stably transfected by Flag-tagged lentivirus with vector, WT DNMT3A or DNMT3A R882C. **(d)** Western blot of DNMT3A and TWIST1 in Kasumi-1 cells stably expressing a non-targeting control shRNA (sh-CTL) or two different shRNA-targeting *DNMT3A* (sh-DNMT3A#1 and #2).

**
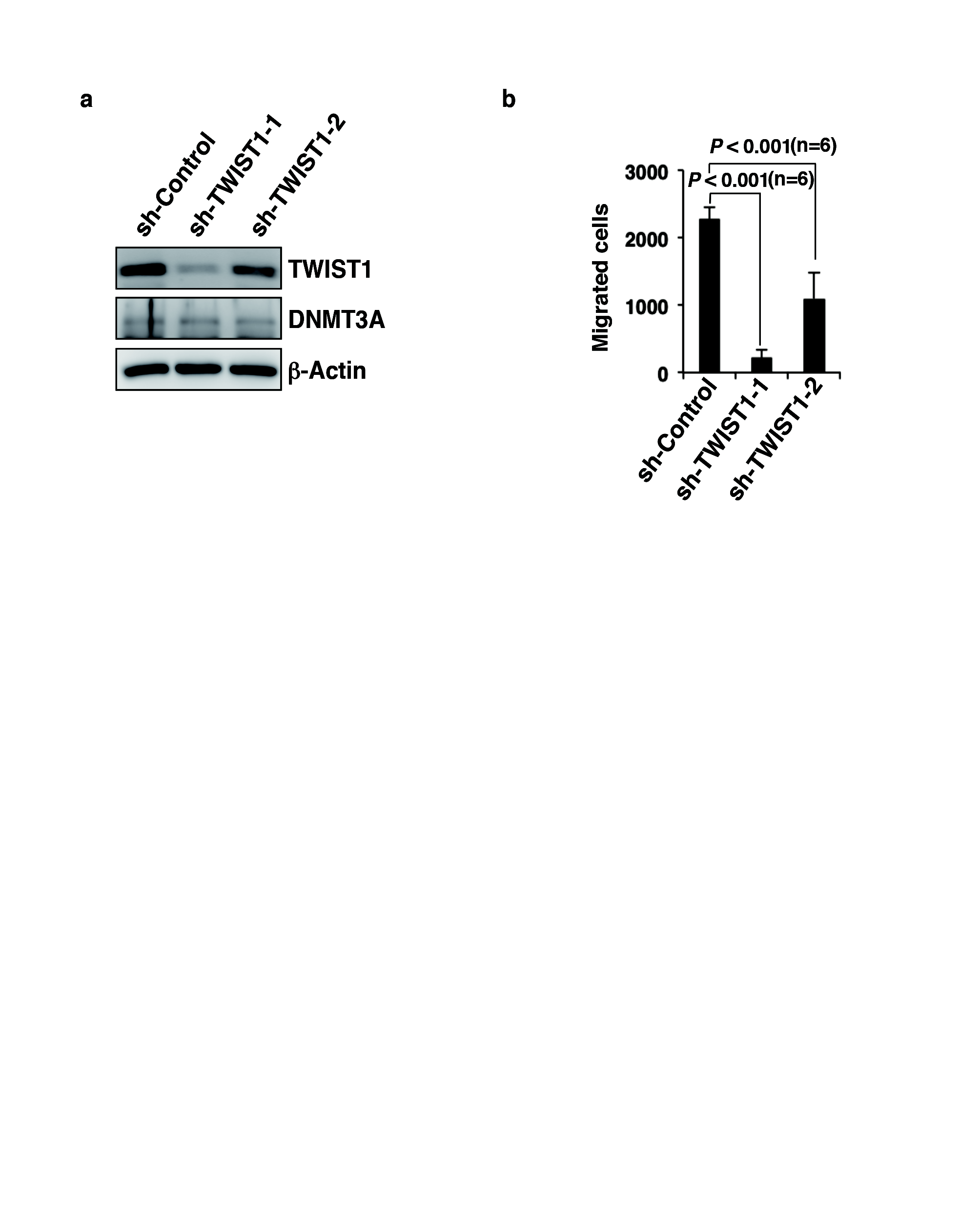
**

**Additional File 1:** **Figure S6 TWIST1 is essential for OCI-AML3 migration.**

**(a)** Western blotting of DNMT3A and TWIST1 in OCI-AML3 strains stably expressing scramble shRNA (sh-control) or two different shRNA-targeting *TWIST1* (sh-TWIST1-1 and -2). **(b)** Transwell assays of OCI-AML3 cells that is stably transfected by lentivirus with sh-control, sh-TWIST1-1 or sh-TWIST1-2. Cells are seeded in a number of 1×10^4^. Data are expressed as mean±SD; n=6 per group.

**
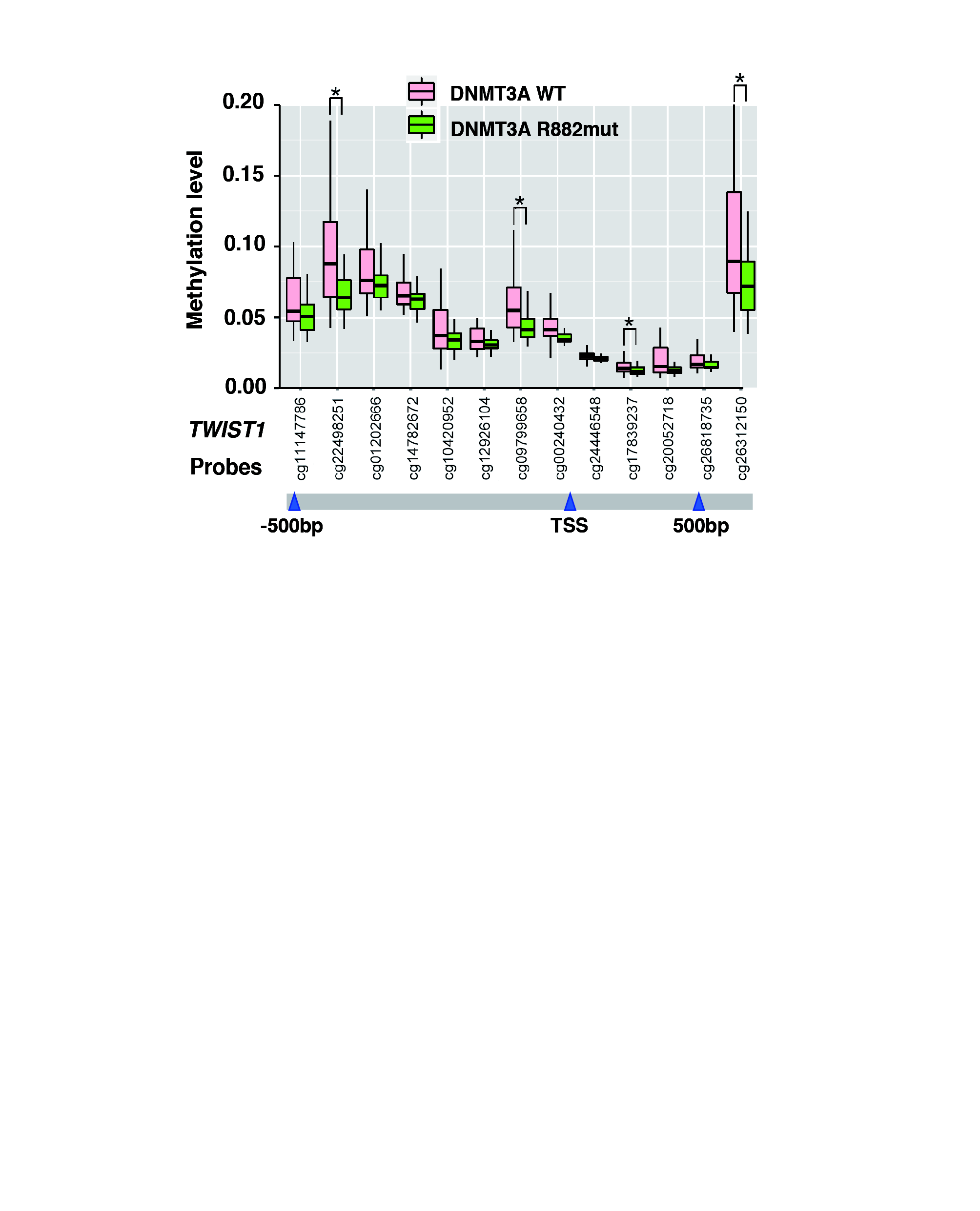
**

**Additional File 1:** **Figure S7 Compare the methylation level of *TWIST1* promoter between patients with WT and mutant DNMT3A.**

DNA methylation data of primary AML samples are from the TCGA database (http://tcga-data.nci.nih.gov/docs/publications/laml_2012/). The methylation levels are analyzed in a region within 500bp up- and downstream of *TWIST1* gene transcriptional start site. Methylation β values are shown for probes associated with *TWIST1*, grouped by *DNMT3A* mutation status (*DNMT3A* WT: 49 samples; *DNMT3A* R882mut: 27 samples). *p* values are calculated by CpGassoc between WT and R882mut samples. The stars represent the significant *p* values (<0.05). Blue arrows indicate the probe positions. TSS: transcriptional start site.
